# In Vivo and In Vitro Grown Lemon-Scented Gum as a Source of Nematicidal Essential Oil Compounds

**DOI:** 10.3390/plants14131892

**Published:** 2025-06-20

**Authors:** Jorge M. S. Faria, Gonçalo Pereira, Ana Cristina Figueiredo, Pedro Barbosa

**Affiliations:** 1 INIAV, I.P., National Institute for Agrarian and Veterinary Research, Quinta do Marquês, 2780-159 Oeiras, Portugal; goncalo.pereira@iniav.pt; 2 GREEN-IT Bioresources for Sustainability, Instituto de Tecnologia Química e Biológica, Universidade Nova de Lisboa (ITQB NOVA), Av. da República, 2780-157 Oeiras, Portugal; 3 CE3C, Centre for Ecology, Evolution and Environmental Changes & CHANGE—Global Change and Sustainability Institute, Faculdade de Ciências da Universidade de Lisboa, DBV, C2, Campo Grande, 1749-016 Lisboa, Portugal; acsf@fc.ul.pt (A.C.F.); pbarbosa@uevora.pt (P.B.); 4 MED, Mediterranean Institute for Agriculture, Environment and Development & CHANGE—Global Change and Sustainability Institute, Institute for Advanced Studies and Research, Évora University, Pólo da Mitra, Ap. 94, 7006-554 Évora, Portugal

**Keywords:** biopesticides, *Bursaphelenchus xylophilus*, citronellal, citronellol, *Corymbia citriodora*, emamectin benzoate, environmental risk analysis, pinewood nematode, sustainable forest management

## Abstract

*Corymbia citriodora* is a eucalypt tree of significant economic value due to its essential oils (EOs), rich in citronellal, citronellol, and other oxygenated monoterpenes with diverse biological activities. Its EOs show potential for the formulation of biopesticides with a lower impact on the environment and human health. This study evaluated the in vitro nematicidal activity of *C. citriodora* EOs, obtained from in vivo and in vitro grown plants, and their main volatile compounds against the pinewood nematode (PWN, *Bursaphelenchus xylophilus*), a major phytosanitary threat. The impact of their main compounds on the environment and human health was assessed using available experimental data and predictions from specialized software. Citronellal and citronellol were the most active EO compounds and exhibited EC_50_ values comparable to the pesticide emamectin benzoate (0.364 ± 0.009 mg/mL). They also displayed superior safety profiles, with reduced environmental persistence and toxicity to non-target organisms. Furthermore, *C. citriodora* shoots were efficiently propagated through an in vitro system and their volatile profile was characterized by a dominance of citronellal (64%), and citronellol (10%), which highlights their potential as a scalable and sustainable source of nematicidal compounds. Remarkably, the EO of *C. citriodora* in vitro shoots was strongly active against the PWN, exhibiting the lowest EC_50_ (0.239 ± 0.002 mg/mL) obtained. These findings underline the viability of *C. citriodora* EOs as a promising alternative for sustainable pest management, addressing the urgent need for environmentally friendly and health-conscious biopesticides while providing a renewable approach to nematode control.

## 1. Introduction

Eucalypts are a diverse group of large trees and shrubs classified within the tribe *Eucalypteae* of the family *Myrtaceae*. This group includes the genera *Eucalyptus*, *Corymbia*, and *Angophora*, which are collectively referred to as gum trees, a name derived from their characteristic sap exudation. Eucalypts are native to Australia and the surrounding regions, and are highly adaptable, thriving in a wide range of climates, which contributes to their cultivation around the globe [1]. They are extensively grown to produce pulp, plywood, and solid wood, with *E. globulus* being the most cultivated species due to its rapid growth and desirable wood properties [2]. In addition, eucalypts are recognized for their essential oils (EOs), which have gained global prominence in the pharmaceutical, cosmetic, and wellness industries. Applications include use in respiratory therapies, topical antiseptics, massage oils, and aromatherapy. Eucalypt EOs are mainly extracted from the leaves and comprise complex mixtures of bioactive compounds, including terpenes and phenylpropanoids, in variable amounts [3]. The highly distinctive and easily recognizable fragrance of eucalypts is largely conveyed by their high content in the oxygen-containing monoterpene 1,8-cineole, commonly known as eucalyptol. However, eucalypts can exhibit a remarkable chemical diversity, allowing for the extraction of a wide range of EO compounds beyond eucalyptol [4,5]. Among the eucalypts, *Corymbia citriodora* (Hook.) K.D. Hill & L.A.S. Johnson, commonly known as lemon-scented gum, holds a prominent place due to its unique aromatic properties. Formerly classified within the genus *Eucalyptus* as *Eucalyptus citriodora* Hook., this species was later reclassified into the genus *Corymbia* based on phylogenetic studies [6]. The EO of *C. citriodora* is characterized by a distinctive lemony aroma, attributed to high levels of the oxygenated monoterpene aldehyde citronellal and the monoterpene alcohols citronellol and isopulegol. The refined EO is widely used in the perfumery industry, where the refreshing lemon fragrance is a much sought-after ingredient in a variety of products, including perfumes, candles, and cosmetics [7]. Additionally, *C. citriodora* EO has demonstrated antimicrobial properties, showing activity against various bacteria and fungi, which supports its use in natural antiseptics and cleaning products [3]. Furthermore, its anti-inflammatory and antioxidant effects have spurred interest in the therapeutic applications of *C. citriodora* EO, particularly in skincare products aimed at reducing oxidative damage [8]. Beyond these applications, it is extensively used as a natural insect repellent, with citronellal acting as the primary bioactive component in pest management [9].

The development of novel active compounds for environmentally safer plant protection products has become an increasingly urgent priority in response to growing concerns about the adverse effects of conventional pesticides. As a result, new-generation pesticides have been introduced, demonstrating promising features such as reduced impacts on non-target organisms and diminished risks to human health. However, despite their improved safety profiles compared to traditional synthetic pesticides, many can still exhibit undesirable toxicological and ecotoxicological properties, highlighting the need for continued refinement and innovation [10]. Even though in the EU, pesticide sales have dropped ca. 10%, the adoption of pest management strategies based on biopesticides is still not very extensive. Against plant parasitic nematodes, phytochemical volatiles, particularly those that compose EOs, have emerged as a compelling alternative, given that they are biodegradable, renewable, and often exhibit potent nematicidal activities while posing reduced risks to human health and the environment [10]. The EOs of a large number of plant species have shown strong nematicidal activities against plant parasitic nematodes, inducing complete mortality at concentrations as low as 2 µg/mL, which underlines their vast potential for the formulation of biopesticides [11]. Beyond the direct nematicidal action, volatile phytochemicals are also valued for their lower persistence in the soil, reducing risks of bioaccumulation and secondary environmental contamination. Regardless of their potential, there are challenges to the widespread adoption of EOs as nematicides, which include variability in chemical composition based on plant origin, seasonal factors, and the scalability and cost of production.

The in vitro production of plant material offers many advantages for obtaining nematicidal metabolites. Compared to in vivo cultivation, in vitro methods significantly reduce the time, space, and resources required for plant propagation [12]. Additionally, in vitro tissue culture allows obtaining plant genetic clones under controlled laboratory conditions, indefinitely, enabling the rapid and large-scale production of plant material as well as a stable metabolite production [12]. In vitro systems enable optimizing the production of volatile phytochemicals in a simplified and reproducible setting, free from microbial contamination. The production of bioactive compounds, including volatiles, can be scaled up using bioreactor systems, providing a sustainable alternative to harvesting plants from natural ecosystems [13,14].

The present work explores the nematicidal activity of *C. citriodora* EO against *Bursaphelenchus xylophilus*, the pinewood nematode (PWN), a destructive phytoparasite affecting Asian and European pine forests. Previous studies have analyzed the nematicidal activity of *C. citriodora* EOs; however, the contribution to the overall activity of their main compounds remains fairly unexplored. In the present work, the main compounds were assayed and compared to the conventional nematicide emamectin benzoate. Their toxicological and ecotoxicological safety was evaluated by comparing experimental data, freely available on online databases, and data estimated by predictive software. Finally, *C. citriodora* in vitro shoot cultures were established, and the volatile profile of their EO was determined and compared to that obtained from its in vivo mature mother plant, and tested against the PWN. The integration of EOs and other phytochemicals into nematode management strategies aligns with the broader goal of developing eco-friendly and sustainable approaches to crop protection.

## 2. Results

### 2.1. In Vitro C. citriodora Shoots

**Figure 1:**
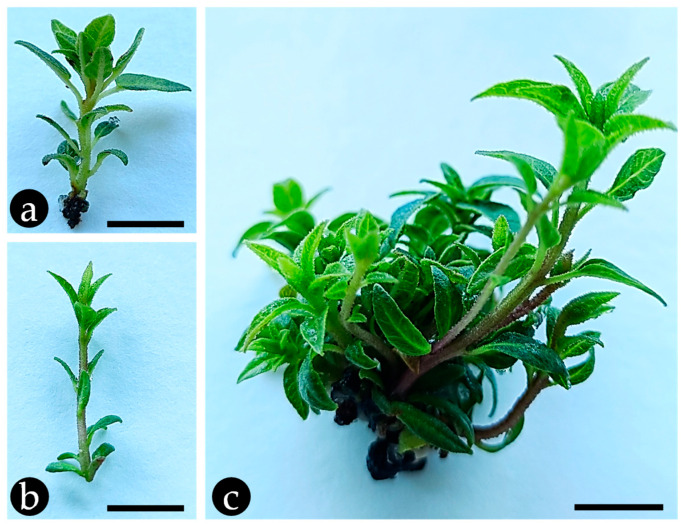
In vitro culture of *Corymbia citriodora* microshoots in Murashige and Skoog (MS) medium [15] supplemented with 8 g/L of agar-agar, 30 g/L of sucrose, 0.5 mg/L of 6-benzylaminopurine (BAP) and 0.1 mg/L indole-3-butyric acid (IBA) [16]. Single microshoots (**a**,**b**) can multiply into shoot clusters with more than 8 microshoots (**c**) after 6 weeks in culture medium. Bar = 0.5 cm.

Shoot cultures were established and maintained to determine if the *C. citriodora* volatile profile would be kept under in vitro conditions, and if their EO would have activity against the PWN. The shoots were obtained by germinating *C. citriodora* seeds in asepsis and transferring the seedling’s upper portion onto Murashige and Skoog (MS) medium [15] supplemented with phytohormones [0.5 mg/L of 6-benzylaminopurine (a cytokinin) and 0.1 mg/L indole-3-butyric acid (an auxin)]. After ca. 8 weeks the first adventitious shoots could be seen emerging. After ca. 4 weeks, shoot clusters were obtained that contained several shoots (ca. 8 ± 2). These were subcultured individually (Figure 1a,b) and formed new shoot clusters after ca. 6 weeks in culture medium (Figure 1c). In vitro lemon-scented gum shoots were kept for over 1 year, using this subculture routine, before experimentation.

The in vitro shoot clusters weighed 419 ± 29 mg (average ± standard error), depending on the number of multiplied shoots (ca. 8 ± 2 per cluster), with individual shoots weighing 48 ± 4 mg. This corresponds to an 8-fold increase in fresh weight over 6 weeks.

### 2.2. Volatile Profile of Corymbia citriodora Essential Oils

The leaves of *C. citriodora* were collected from a mature 10-year-old tree, and the EO was extracted through hydrodistillation using a Clevenger apparatus. The yield of the EO obtained was 0.85% (volume/fresh weight). The volatile profile of the EO was analyzed and found to consist primarily of terpenes, namely, 88% monoterpenes (84% of which were oxygen-containing monoterpenes) and 9% sesquiterpenes (Table 1).

In the *C. citriodora* EO, the monoterpene aldehyde citronellal was the dominant compound (38%); followed by isopulegol (14%) and citronellol (13%), both monoterpene alcohols; and 1,8-cineole (12%), a monoterpene ether (Table 1). The sesquiterpene hydrocarbon *trans*-β-caryophyllene, and monoterpene citronellyl acetate were also detected in considerable amounts (7 and 3%, respectively).

The EO of the in vitro *C. citriodora* shoot clusters was also obtained and compared to its in vivo counterpart. The yield of the EO obtained was 0.25%. The volatile profile of the shoot clusters EO was seen to be only slightly different from that obtained for the in vivo tree EO (Table 1). Citronellal dominated the volatiles of the in vitro shoot cultures, 64%, which is almost twice that obtained for the in vivo leaves EO. Other dominant volatiles, e.g., 1,8-cineole or isopulegol, showed lower proportions than in the mature plant EO, while compounds such as citronellol, citronellyl acetate, or *trans*-β-caryophyllene continued to be present in relatively high proportions (Table 1).

### 2.3. Nematicidal Activity Against the Pinewood Nematode

*Corymbia citriodora* EO was screened against the PWN alongside its main compounds with relative amounts above 5%, namely, citronellal, citronellol, 1,8-cineole, isopulegol, and *trans*-β-caryophyllene, in comparison to the commercial nematicide emamectin benzoate. At the highest concentration tested, the pure *C. citriodora* EO induced a corrected mortality of 98.1 ± 0.2%, while 1,8-cineole, isopulegol, and *trans*-β-caryophyllene caused lower mortalities, 8.5 ± 0.2, 22.9 ± 0.9, and 17.9 ± 0.6%, respectively (Table 2). Conversely, citronellal, citronellol, and the pesticide emamectin benzoate caused complete mortality (100 ± 0.0%) to the PWN. The EO obtained from in vitro shoots of *C. citriodora* also induced 100% mortality to the PWN at the highest concentration tested (2 mg/mL).

**Figure 2:**
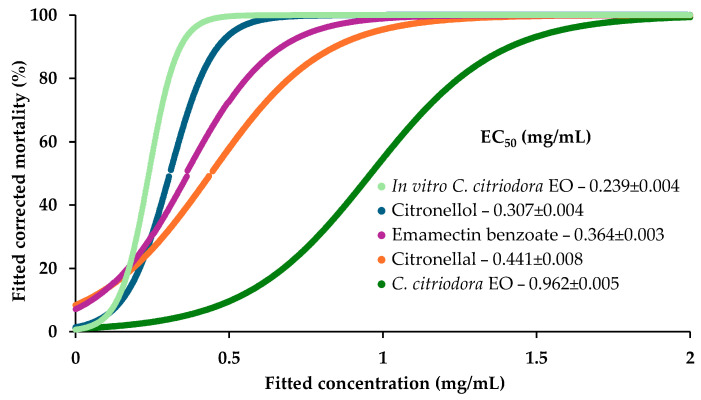
Dose–response curves fitted to the pinewood nematode corrected mortality (%) values plotted against the concentration (mg/mL) values of *Corymbia citriodora* (dark green) and in vitro *C. citriodora* shoot essential oils (light green) or its main compounds, the alcohol citronellol (blue) and its aldehyde citronellal (orange), in comparison to the conventional pesticide emamectin benzoate (purple). The half-maximal effective concentration (EC_50_) values were determined for each based on the equation for the fitted dose–response curves.

The EOs of *C. citriodora*; in vitro *C. citriodora* shoots; and citronellal, citronellol, and emamectin benzoate were screened against the PWN at lower concentrations to characterize their toxicological profiles. The highest half maximal effective concentration (EC_50_) value was determined for *C. citriodora* EO, namely 0.962 ± 0.005 mg/mL, followed by 0.441 ± 0.008 mg/mL for citronellal, 0.364 ± 0.009 mg/mL for emamectin benzoate, and 0.307 ± 0.004 mg/mL for citronellol (Table 3, Figure 2, Supplementary Table S1). The EO obtained from the in vitro *C. citriodora* shoots reached the lowest value (0.239 ± 0.004 mg/mL), suggesting a high activity against the PWN and a potential synergistic interaction between compounds at their relative amounts.

The values of EC_20_ were higher for the *C. citriodora* EO (0.677 ± 0.003 mg/mL) followed by citronellol (0.209 ± 0.002 mg/mL), citronellal (0.186 ± 0.004 mg/mL), the in vitro *C. citriodora* shoot EO (0.174 ± 0.009 mg/mL) and, finally, emamectin benzoate (0.170 ± 0.006 mg/mL). For the EC_80_ values, the sequence from highest to lowest was *C. citriodora* EO > citronellal > emamectin benzoate > citronellol > in vitro *C. citriodora* shoot EO (Table 3). The values of EC_100_ were higher for emamectin benzoate (2.0), followed by *C. citriodora* EO (1.8), citronellal (1.7), citronellol (1.3), and lastly by in vitro *C. citriodora* shoot EO (0.9).

### 2.4. Potential for Higher Environmental Safety

The potential environmental impacts of *C. citriodora* EO or its main nematicidal compounds were assessed by comparing their predicted environmental parameters and reported toxicity thresholds for aquatic model organisms with those of the pesticide emamectin benzoate. The predicted environmental distribution was estimated for each compound based on its chemical properties. While emamectin benzoate showed a higher potential affinity to the soil environmental compartment, citronellal and citronellol were predicted to allocate more pronouncedly to the air (69 and 26%, respectively) and water (13 and 61%, respectively) environmental compartments, and less to the soil (18 and 12%, respectively) (Table 4). Compound persistence was also predicted to be 30- to 40-fold higher for emamectin benzoate.

The predicted compound volatilization from aquatic environments was also seen to be very different between the EO compounds and emamectin benzoate. Compound half-life values for the river model were predicted to be much lower for citronellal and citronellol (4 and 14 h, respectively) than for the conventional pesticide (more than 100 years), given that emamectin benzoate is non-volatile. The same tendency was observed for the lake model (Table 4). In the aquatic environment, the estimates for bioaccumulation were again highest for emamectin benzoate. The projected bioaccumulation and bioconcentration values were, for emamectin benzoate, 355- and 73-fold higher than those of citronellal, respectively, and 433- and 88-fold higher than those of citronellol, respectively. The biotransformation estimates, given in compound half-life, were 59 days for emamectin benzoate, while for citronellal and citronellol only 22 and 12 h (Table 4). Due to its chemical properties, emamectin benzoate was predicted to be readily removed through wastewater treatment procedures, being mainly adsorbed to the solid sludge (93%). For citronellal and citronellol, removal was predicted to be lower (16 and 8%, respectively), undoubtedly due to their affinity to water or air.

The toxicity thresholds reported for model organisms of different aquatic trophic levels also favored the phytochemicals citronellal or citronellol in comparison to the pesticide emamectin benzoate. The acute toxicity threshold (LC_50_) values reported for fish were 110- and 74-fold higher for citronellal and citronellol, respectively (Table 5). This difference was even higher for the model organisms for algae and invertebrates. For algae, the reported toxicity thresholds for citronellal and citronellol were 13,300- and 2400-fold higher, respectively, than that of emamectin benzoate; while for invertebrates this difference was 1243- and 2500-fold higher for citronellal and citronellol, respectively (Table 5).

### 2.5. Predicted Lower Impacts for Human Health

To assess the potential advantages to human health of using *C. citriodora* EO and/or its main nematicidal compounds in the formulation of biopesticides against plant parasitic nematodes, the experimental acute toxicity thresholds reported on online databases were retrieved and compared with those reported for the conventional pesticide emamectin benzoate. For both oral and dermal routes of exposure, the toxicity thresholds of citronellal or citronellol were much higher than emamectin benzoate. The LD_50_ value for the oral acute toxicity threshold of emamectin benzoate was 30- and 42-fold lower than those reported for citronellal or citronellol, respectively (Table 6). For the dermal acute toxicity thresholds, emamectin benzoate was reported to show an LD_50_ value ca. 6-fold lower than both citronellal and citronellol.

To gauge the potential impacts on human health of using *C. citriodora* and/or its main nematicidal compounds as alternatives to the pesticide emamectin benzoate, the ADMETlab 3.0 online tool was used to predict several toxicological endpoints. The software assigns a number between 0 and 1 to each compound and for each endpoint, which symbolizes the probability of the compound possessing toxicity, with 0 being the least probable and 1 being the most probable. Essentially, compounds assigned with values above 0.7 are very likely to exhibit toxicity. As before, emamectin benzoate was predicted to have a different behavior than citronellal or citronellol. For these, the predicted toxic effects were mainly related to skin sensitization or eye irritation. However, emamectin benzoate showed a high probability of having a very low toxic dose threshold in humans, estimated through the software’s FDA (U.S. Food and Drug Administration) maximum (recommended) daily dose (FDAMDD) endpoint. This pesticide also showed a high probability of displaying toxicity by promoting genomic instabilities and/or epigenetic alterations, which translate into a variety of diseases (genotoxicity), drug-induced liver injury, and drug-induced neurotoxicity, which leads to neurological symptoms and episodes resembling psychosis, or ototoxicity, which is harm to the inner ear by either damaging the ear’s structures directly or the nervous system.

## 3. Discussion

The development of novel biopesticides has become critical as the detrimental impacts of conventional pesticide overuse are being progressively revealed. Extensive and indiscriminate application of synthetic pesticides has led to persistent residues in ecosystems, resulting in significant damage to biodiversity, including the disruption of non-target organism populations and their essential ecological functions [25]. Furthermore, these practices have accelerated the evolution of pesticide resistance among pest populations, undermining long-term pest management strategies and posing substantial challenges to agricultural sustainability [25]. In this context, biopesticides, derived from natural biological sources, can offer a promising alternative. *Corymbia citriodora* is a fast-growing tree species extensively cultivated for its high-quality timber. Its wood, characterized by its hardness and durability, is widely employed in heavy-duty construction projects and as a sustainable source of fuel [26]. In addition to its timber value, *C. citriodora* exhibits a substantial EO production, as the smaller shoots and leaves—often considered by-products—can yield a high content of EO, reaching up to 2% by weight [27]. This high yield, coupled with its chemical composition, puts *C. citriodora* as a promising candidate for the development of bio-based nematicides. The EO of *C. citriodora* analyzed in this study exhibited a chemical profile dominated by the oxygenated monoterpenes citronellal, citronellol, 1,8-cineole, and isopulegol, which collectively accounted for approximately 80% of the total EO composition. This chemical profile is typical for *C. citriodora* EOs obtained in several parts of the world, albeit with variations in compound proportions [7]. At the highest concentration tested (2 mg/mL), *C. citriodora* EO showed a strong nematicidal activity against the PWN. Moreover, when tested solely, its main components citronellal or citronellol induced complete mortality (100%), while 1,8-cineole or isopulegol displayed only marginal nematicidal activity under the same experimental conditions, highlighting the dominant role of citronellal and citronellol in the EO’s overall nematicidal strength. Although these compounds exhibited notable nematicidal activities individually, the observed overall bioactivity of the complete EO could not be entirely attributed to their simple additive effect, in their respective relative proportions. This suggests that synergistic interactions between EO components may play a critical role in enhancing their efficacy. This activity may arise from complex biochemical and biophysical interactions among the compounds, which can influence penetration into nematode cuticles, target-site binding, or interference with nematode physiological pathways [28,29,30].

In other studies, *C. citriodora* EOs were reported to demonstrate varying levels of nematicidal activity against the PWN. Reported activity ranges from less than 40% mortality, at a concentration of 2 mg/mL, to as high as 73% mortality at 10 mg/mL [29,31,32]. These differences in activity can be attributed not only to the concentration of the EOs applied but also to the choice of solubilizing agent, which influences their dispersion and bioavailability [33]. Most critically, the nematicidal potential can be strongly linked to the specific chemical composition of the EOs, which was not detailed in some works, but is known to vary depending on factors such as geographic origin, plant growth conditions, and extraction methods [31,32,34]. *C. citriodora* EOs have demonstrated significant efficacy against other plant-parasitic nematodes, namely the root-knot nematodes, which are among the most destructive agricultural pests globally. For instance, complete mortality of second-stage juveniles (J2) of *Meloidogyne incognita* was observed at a concentration of 0.25 mg/mL [35]. In other studies, remarkably low lethal concentration values (LC_50_) have been reported over a 24 h exposure period, such as 2.4 µg/mL [36] or 746 µg/mL [37], for EOs characterized by an exceptionally high citronellal content, ranging from 82% to 84%, which likely contributes to their enhanced nematicidal properties. In fact, in the present study, the nematicidal strength of citronellal (0.441 ± 0.008 mg/mL) seems to be only slightly lower than emamectin benzoate (0.364 ± 0.009 mg/mL), a conventional pesticide. Citronellol stood out for the low EC_50_ value, 0.307 ± 0.004 mg/mL, showing very good properties against the PWN. In a previous study, the different nematicidal potencies of citronellal and citronellol were also reported. Citronellal was reported to exhibit an EC_50_ of 0.245 mg/mL, 0.235 mg/mL, and 0.169 mg/mL against the male, female, and juvenile stages of the PWN, respectively. In contrast, citronellol demonstrated a lower nematicidal potency, with EC_50_ values of 0.187 mg/mL, 0.139 mg/mL, and 0.253 mg/mL against the same PWN life stages [38]. These findings highlight significant variations in the nematicidal activities of these two compounds, with citronellol showing superior efficacy against adults rather than juveniles, but citronellal displaying stronger effects against juveniles. This disparity in activity suggests that the developmental stage of the nematode plays a critical role in modulating the response to these compounds, a factor that may have influenced the outcomes observed in the present study, since the suspensions used were composed of mixed life stages of the PWN.

In terms of their environmental impacts, citronellal and citronellol are predicted to have more favorable environmental profiles compared to emamectin benzoate. Specifically, these compounds exhibited a higher predicted affinity for the air and water environmental compartments, in contrast to the strong affinity of emamectin benzoate for the soil compartment. Additionally, the predicted environmental persistence for emamectin benzoate is up to 40-fold higher than that of citronellal and citronellol. The environmental implications of this difference can be significant. While a high affinity for air is generally less hazardous, the increased affinity of citronellal and citronellol for the water compartment can be of greater concern. This suggests that these compounds may be more readily available for aquatic organisms, thereby posing a potential risk to aquatic ecosystems. However, it is important to note that citronellal and citronellol are predicted to volatilize from water bodies much more rapidly than emamectin benzoate. This could mitigate their environmental impact in aquatic environments to some extent, as their persistence in water may be significantly shorter than that of emamectin benzoate. This tendency is further supported when examining the respective bioaccumulation and bioconcentration factors for each compound. Citronellal and citronellol exhibited lower values than emamectin benzoate, suggesting a reduced potential for bioaccumulation in aquatic organisms. Additionally, the likelihood of biotransformation in an aquatic context appears to be higher for citronellal and citronellol, further diminishing their persistence and toxicity in aquatic environments. This comparison is particularly significant when evaluating the acute toxicity thresholds reported for citronellal or citronellol relative to those for emamectin benzoate. For model organisms of fish, algae, and invertebrates, the LC_50_/EC_50_ values for emamectin benzoate are markedly lower than those of citronellal and citronellol. This indicates that emamectin benzoate can exhibit substantially higher acute toxicity across these groups. Such findings emphasize the potential ecological risks associated with the use of emamectin benzoate, particularly when it is released into aquatic environments. Conversely, the predicted removal of emamectin benzoate through wastewater treatment processes appears to be significantly more effective compared to that of citronellal or citronellol. This discrepancy is likely attributable to the distinct physicochemical properties of emamectin benzoate, particularly its higher molecular weight and probable greater affinity or favorable adsorption onto soil or sludge matrices. In contrast, the lower molecular weight and reduced adsorption affinity of citronellal and citronellol may limit their retention in solid fractions, thereby reducing the overall efficiency of their removal. In fact, citronellal is approved for use in a wide range of products, including air fresheners, cleaning agents, and floor polishes [39]. The International Fragrance Association (IFRA) classifies citronellal as non-persistent, non-bioaccumulative, and non-toxic, signifying that it degrades readily in the environment, does not accumulate in living organisms, and poses minimal ecological toxicity [40]. Its compliance with IFRA Environmental Standards reflects comprehensive testing, confirming its safety for inclusion in fragrance formulations.

From a human health safety perspective, citronellal and citronellol demonstrate significantly higher acute toxicity thresholds compared to emamectin benzoate based on data from both oral and dermal exposure routes. According to the Globally Harmonized System of Classification and Labelling of Chemicals (GHS), emamectin benzoate is categorized as a Class 3 toxicant. This designation applies to substances with oral LD_50_ values ranging from 50 to 300 mg/kg or dermal LD_50_ values between 200 and 1000 mg/kg, reflecting a moderate level of acute toxicity. In contrast, citronellal and citronellol are classified as Class 5 molecules, which include compounds with oral or dermal LD_50_ values exceeding 2000 mg/kg [41]. This classification represents the lowest acute toxicity category under the GHS framework, indicating a significantly reduced risk of adverse effects upon single-dose exposure. The substantial difference in toxicity levels highlights the comparatively safer toxicological profile of citronellal and citronellol. This distinction in toxicity also extends to the predicted toxicity endpoints for these compounds. Citronellal and citronellol, despite their lower acute toxicity (Class 5 toxicants under the GHS), may still exert mild adverse effects on external parameters, such as skin sensitization or eye irritation. These effects, while noteworthy, are generally localized and reversible, and are consistent with their widespread use in cosmetics, fragrances, and other consumer products where such risks are considered manageable through proper formulation and labeling [42]. In contrast, emamectin benzoate, classified as a Class 3 toxicant, is associated with a broader spectrum of potential systemic toxicity endpoints. These include genotoxicity, drug-induced liver injury, drug-induced neurotoxicity, and ototoxicity. Genotoxicity refers to the compound’s potential to damage genetic material, raising concerns about long-term carcinogenic risks [43]. Drug-induced liver injury suggests hepatotoxic effects, which could result in metabolic disturbances or hepatic dysfunction with prolonged or high-dose exposure. Neurotoxicity and ototoxicity further highlight emamectin benzoate’s potential to disrupt the central nervous system or auditory function, respectively, underscoring its comparatively higher risk profile [44]. The divergence in predicted toxicity endpoints reflects fundamental differences in the chemical properties, mechanisms of action, and biological interactions of these compounds. While citronellal and citronellol may require attention to mitigate minor irritative effects in specific applications, their lack of systemic toxicities such as genotoxicity or organ-specific damage makes them safer candidates for human exposure. Conversely, emamectin benzoate’s more severe toxicity endpoints necessitate stricter regulatory controls, risk assessments, and limitations on its usage to minimize human health risks. These findings reinforce the potential of citronellal and citronellol as safer alternatives in contexts requiring low-toxicity agents, particularly when compared to substances like emamectin benzoate.

Overall, the ecotoxicological characteristics of the main compounds of *C. citriodora* EO emphasize its relevance as a valid candidate for safer pest management strategies. Compared to emamectin benzoate, it can exhibit lower environmental persistence, a reduced bioaccumulation potential, and a greater tendency to volatilize, collectively supporting a diminished risk to non-target ecosystems. The toxicological data further indicate substantially higher acute exposure thresholds and a potential absence of systemic toxic effects, such as genotoxicity or neurotoxicity, thereby supporting a more favorable safety profile for human exposure. Notably, certain constituents have exhibited very strong nematicidal activity, underscoring the potential of *C. citriodora* EO and its main components as effective, environmentally benign alternatives to conventional synthetic pesticides.

Interestingly, the in vitro culture of *C. citriodora* shoots yielded volatile profiles dominated by citronellal (64%), with a lower chemical complexity compared to the EO extracted from the mature plant. The distinctive lemon-like scent of *C. citriodora* was immediately notable in the cultured explants; this is likely attributable to the high density of trichomes, suggesting a resemblance to the juvenile leaf stage. In eucalypt species, pronounced leaf dimorphism is evident between juvenile and mature leaves. Juvenile leaves, observed in seedlings, are oval to round, occasionally sessile, glaucous, and densely covered with soft trichomes. In contrast, the leaves of mature trees are alternate, entire, petiolate, and lanceolate, with a thick, stiff, highly cutinized, and coriaceous structure, reflecting adaptations to reduce water loss and withstand environmental stressors [45]. Remarkably, the EO obtained from *C. citriodora* in vitro shoots showed stronger activity against the PWN than the in vivo *C. citriodora* EO or the main pure compounds tested. This suggests that at their respective proportions in the EO, its main compounds may interact synergistically against the PWN. These types of interaction have previously been seen for monoterpenes, such as geraniol, against plant parasitic nematodes and must definitely exist between other monoterpenes and sesquiterpenes [28,46]. The in vitro system demonstrated the potential for rapid and scalable production of nematicidal volatiles, with an 8-fold increase in biomass after 6 weeks in culture. In other studies involving *C. citriodora*, in vitro shoot culture has been identified as an effective method for the rapid propagation of this economically significant species [47]. While in vitro shoot culture remains a relatively costly technique and may not be suitable to produce low-cost compounds, it offers notable advantages. These include consistent, year-round production—an essential feature for industrial applications—as well as the potential for optimization to meet specific objectives and enhance targeted traits. Nevertheless, the use of EOs as biopesticides in agricultural applications still presents several limitations. Mainly, they exhibit rapid degradation when exposed to environmental factors such as sunlight, air, and moisture, resulting in short residual activity and the need for frequent reapplication. Additionally, some EOs can be phytotoxic at higher concentrations, potentially harming the crops they are intended to protect. Furthermore, the lack of standardized regulatory frameworks can hinder their integration into pest management programs.

Even though our study showcases the promising nematicidal potential and favorable environmental and toxicological profile of *C. citriodora* EO and its main components, key gaps can be highlighted. For instance, the molecular mechanisms responsible for nematicidal activity or the interactions among EO constituents are not fully understood and warrant further investigation using, for example, transcriptomic and/or metabolomic approaches. Future studies must aim for field-level assessments, necessary to validate laboratory efficacy and assess real-world environmental fate, phytotoxicity, and non-target effects. Also, establishing standardized formulations and exploring encapsulation or slow-release technologies may help overcome the current limitations in EO stability. These approaches could greatly facilitate the practical integration of *C. citriodora* EO into sustainable pest management frameworks.

## 4. Materials and Methods

### 4.1. Chemicals

Pure analytical standards of *C. citriodora* EO main compounds (present in the EO above 5%) citronellal (purity 96%), citronellol (purity ≥ 95%), 1,8-cineole (purity 99%), isopulegol (purity ≥ 98%), and *trans*-β-caryophyllene (purity ≥ 98%) were acquired from Sigma-Aldrich (St. Louis, MO, USA). The HPLC-grade solvent methanol (purity 99.9%), used for stock solutions, was acquired from Fisher Chemicals (Portsmouth, NH, USA). The commercial nematicide emamectin benzoate (Pursue^®^, Syngenta, Lisbon, Portugal) was also tested.

### 4.2. Plant Material, Essential Oil Isolation and Analysis

Fresh lemon-scented gum shoots, with mature leaves, and seeds were collected, in June 2023, in the vicinity of Loures, Lisbon, Portugal, from the middle section of the main shoot of a 10-year-old *C. citriodora* tree. The EOs were obtained by the hydrodistillation of three replicates of the fresh shoots for 3 h according to the European Pharmacopoeia [48] and analyzed with gas chromatography coupled with flame ionization detector (GC-FID) for quantification and by gas chromatography coupled with mass spectrometry (GC-MS) for component identification [49]. Briefly, the GC-FID analyses were run on a PerkinElmer AutoSystem 9000 gas chromatograph (PerkinElmer, Shelton, CT, USA), equipped with two flame ionization detectors, with a data handling system and a split–splitless injector port into which two columns of different polarities were inserted: a DB-1 fused-silica column (polydimethylsiloxane, 30 m × 0.25 mm i.d., film thickness 0.25 μm; J & W Scientific Inc., Rancho Cordova, CA, USA) and a DB-17HT fused-silica column [(50% phenyl)-methylpolysiloxane, 30 m × 0.25 mm i.d., film thickness 0.15 μm; J & W Scientific Inc.]. The oven temperature was programmed at 3 °C/min from 45 to 175 °C, then at 15 °C/min up to 300 °C, and finally held isothermal for 10 min. The temperatures of the injector and detector were 280 °C and 300 °C, respectively. The carrier gas was hydrogen (30 cm/s). The split sampling technique ratio was 1:50. The injection volume was 0.1 μL of a 1:1 distilled *n*-pentane-EO solution. The EO percentage composition was determined using the normalization method for the GC peak areas, calculated as the average of two injections per sample, without using the response factors.

The GC-MS analyses were performed on a PerkinElmer Clarus 600 gas chromatograph, equipped with a DB-1 fused-silica column as described above, and interfaced with a PerkinElmer 600T mass spectrometer (software version 5.4.2.1617, PerkinElmer, Shelton, CT, USA). Oven and injector temperatures were the same as for the GC analyses. Transfer line at 280 °C. Carrier gas, helium (30 cm/s). Ion source at 220 °C. Split ratio, 1:40. Ionization energy, 70 eV. Scan range at 40–300 u and scan time of 1 s. Component identity was established by the comparison of their retention indices relative to *n*-alkane (Sigma) indices and GC-MS spectra from a laboratory library generated with commercially available standards, laboratory-synthesized components, and laboratory-isolated compounds.

### 4.3. Establishment of C. citriodora In Vitro Shoot Cultures

In vitro shoot cultures were established from aseptic *C. citriodora* seedlings. The seeds were obtained from a 10-year-old tree in 2023, and then cleaned by placing ca. 20 in 5 mL microtubes and washing vigorously with tap water for 5 min, followed by adding 3 mL of a commercial detergent solution (10 drops per 100 mL distilled water), mixed vigorously for 10 min, and dipped in an ultrasonic bath for 5 min. Afterward they were rinsed 3× with running tap water and surface sterilized by adding 3 mL of ethanol (96%) to the microtubes, mixing vigorously, and dipping in an ultrasonic bath for 10 min. Under a flow hood, the seeds were washed 3× with 3 mL of sterile distilled water. The seeds were maintained in darkness at 24 ± 1 °C until germination. The seedlings were then transferred to a growth chamber at 24 ± 1 °C and 16 h light. The shoot cultures were initiated by sectioning the upper portion (hypocotyl and cotyledon) of each seedling and separately inoculating in a semi-solid multiplication medium consisting of Murashige and Skoog (MS) medium [15] supplemented with 30 g/L of sucrose, 0.5 mg/L of 6-benzylaminopurine (BAP), and 0.1 mg/L indole-3-butyric acid (IBA) [16]. BAP is a synthetic cytokinin widely used to induce shoot proliferation due to its activity in promoting multiplication in plant cells, while IBA is an auxin that promotes cell elongation, among other effects, in plant tissue. The balance in their concentration in the culture medium allows for controlling shoot proliferation and elongation. The concentrations used in this study were previously seen to induce substantial shoot proliferation without promoting excessive miniaturization in another woody plant (pine) [16]. MS medium is widely used for plant tissue culture due to supporting the growth of a broad range of plant species. The pH was set to 5.8 before the addition of 0.8% (*w*/*v*) agar and autoclaved at 121 °C for 15 min. Routine subculture of *C. citriodora* microshoots was performed after ca. 6 weeks in SacO2 ^®^ (Ghent, Belgium) microboxes [9.7 cm base diameter per 8 cm height and green filter (XXL+) on the lid for air exchange] containing ca. 100 mL of culture medium [16]. The shoot cultures in multiplication medium were kept under controlled environmental conditions of 16 h of light at 24 ± 1 °C and 8 h of darkness at 18 °C. This subculture routine was used to maintain the in vitro lemon-scented gum shoots for over 1 year before testing. Fresh weight was determined by weighing clusters and individual shoots after a 6-week subculture period (6 replicates for each). To obtain in vitro *C. citriodora* EO, the microshoot clusters grown in 7 to 8 microboxes were pooled for hydrodistillation. Three replicates of the pooled microshoots were extracted and analyzed.

### 4.4. In Vitro Culturing of the Pinewood Nematode

Suspensions of the PWN were obtained from the isolate Bx0.13.003 [50] kept as a reference isolate at the Plant Nematology Lab of the National Institute for Agrarian and Veterinary Research (INIAV, I.P.) at Oeiras, Portugal. This isolate was grown in vitro by adding 1 mL of a mixed life-stage PWN suspension (1000 PWNs) to an axenic culture of a non-sporulating strain of the fungus *B. cinerea*. This strain produces solely mycelium, on which the PWN feeds abundantly, and allows obtaining large amounts of the nematode. The fungal axenic cultures were previously established on steam-sterilized hydrated barley grains (*Hordeum vulgare* L.) (ca. 15 g cereal/15 mL ultrapure water in 250 mL Erlenmeyer flasks) kept for 7 to 10 days at 25 ± 1 °C [29]. Following inoculation with the PWN, the cultures were kept in darkness for 7 to 10 days until the fungal mat was consumed by the nematode population, and then set in a modified Baermann funnel, for 24 h, to obtain fresh PWN suspensions [51], used for the direct-contact assays, for further inoculations, or to be stored at 11 °C. An Olympus SZX12 (Olympus, Tokyo, Japan) stereomicroscope (40×) was used for the assessment of PWN numbers and/or survival rates.

### 4.5. Screening the Activity of C. Citriodora Essential Oil and Main Compounds

The nematicidal activity of the EOs of lemon-scented gum, in vitro *C. citriodora* shoots, its main compounds (≥5%), and the pesticide emamectin benzoate was assessed through direct-contact bioassays. In each well of a flat-bottom 96-well microtiter plate (Carl Roth GmbH & Co. KG, Karlsruhe, Germany), 95 µL of an aqueous suspension containing 80 to 100 mixed life-stage PWNs was added to 5 µL of an EO/compound stock solution, prepared in HPLC-grade methanol at 40 mg/mL, to obtain a final concentration of 2 mg/mL [50]. Lower concentrations were obtained by serial dilutions with a dilution factor of two for the final concentrations of 1, 0.5, 0.25, and 0.125 mg/mL per well. The range in concentrations selected was based on previous work performed on the activity of EOs on the PWN [50]. Five µL of ultrapure water (blanks) or five µL of methanol (control) were also tested to determine natural mortality or to understand the contribution of methanol to PWN mortality. The plates were then sealed with plastic film to reduce EO escape through volatilization and mixed in an orbital shaker (IKA labortechnik, Staufen, Germany) at 800 r.p.m. for 1 min. The plates were then covered with aluminum foil and kept at 25 ± 1 °C in an orbital shaker at 50 r.p.m. After 24 h, live and dead PWNs were recorded under a stereomicroscope (40×). Motionless PWNs were considered dead if they remained unresponsive after physical prodding. Three separate trials were performed for each sample in a total of 10 bioassays (replicates).

### 4.6. Ecotoxicological Endpoints of Environmental Safety

To gauge the potential environmental safety of a compound or compound formulation, several sources of information were used. In silico models can help in predicting important environmental parameters such as the distribution and/or fate of a compound based on its known chemical properties. Although there are concerns regarding the limitations of using fugacity models in accurately reflecting what occurs in natural conditions, they can still provide an important indication of the potential of each compound to occupy the environmental compartments. To understand the potential environmental dispersion of a large amount of citronellal, citronellol, or the pesticide emamectin benzoate, for example, in a hypothetical spillage, their predicted environmental distribution (PED) percentages were determined through an equilibrium criterion model [52]. For this effect, the Level I Mackay Fugacity Model beta version 4.39, made available by Trent University, Canada [53], was set to predict a situation in which a fixed 100,000 kg compound quantity was introduced to a closed system under steady-state and equilibrium conditions at 25 °C. To complete the computation of PED percentages, the chemical properties of the EO compounds and emamectin benzoate were retrieved from the PubChem [20] and the PPDB—Pesticide Properties Database [21] online databases (Table 7). These were physical and chemical properties, namely, the molecular mass (g/mol), melting point (°C), vapor pressure (Pa), and solubility in water (mg/L), as well as the partition coefficients air/water partition, Henry’s law constant, (Pa.m^3^/mol), *n*-octanol/water partition (log value of Kow), and soil organic carbon/water partition (Koc). Additionally, the EPISuite™ software (version 4.11) [18], which is freely available from the US Environmental Protection Agency, was used to predict the persistence of a compound (in h) and its potential volatilization rate from a river or lake model (half-life in h) using the WVOLWIN™ module; estimate the bioaccumulation factor (BAF) for generic fish species in the upper trophic level of aquatic food webs, the bioconcentration factor (BCF) for water exposure, and biotransformation (half-life in days) in an aquatic environment, based on the Arnot–Gobas method [19], using the BCFBAF™ module; and predict the percentage removal of a chemical in a typical activated sludge-based sewage treatment plant using the STPWIN™ module.

Free online databases also gather experimental data obtained from acute toxicity tests performed on model species of important organism groups in several ecosystems, which provides a solid approach to the assessment of the impacts on biodiversity. For EU chemical legislation, testing acute aquatic toxicity is a basic requirement, and it is usually performed through the short-term exposure of the organism to a series of concentrations of a target substance that yields an EC_50_ value. The European Chemicals Agency (ECHA) [22], the PPDB (the Pesticide Properties Database) [21], and PubChem [20] databases were used to compile information on the acute toxicity thresholds for fish, algae, and invertebrate model organisms.

### 4.7. Toxicological Endpoints of Concern to Human Health

To understand the benefits to human wellbeing of using *C. citriodora* EO or its main compounds in comparison to the pesticide emamectin benzoate, several experimental and predictive endpoints were listed. For citronellal, citronellol, and the pesticide emamectin benzoate, important toxicological parameters have been screened and reported. The acute oral toxicity threshold is the dose of a compound administered orally that is lethal to 50% of the tested population (LD_50_). On the other hand, the acute dermal toxicity threshold is the lethal dose (LD_50_) that causes toxic effects through skin exposure to 50% of the tested population. For the present work, acute oral and dermal toxicity thresholds were retrieved from the European Chemicals Agency (ECHA) [32], the PPDB (the Pesticide Properties Database) [33,34], and PubChem [35] free online databases.

For difficult-to-determine toxicity endpoints, such as those related to toxicity to human organ functions, computational toxicity estimations are very helpful, being faster than the determination of toxic doses in animals and helping reduce experiments with animals. The harmful effects on human health can be gauged by resorting to specialized software, such as the webtool ADMETlab 3.0 [23], which can estimate the probability of a compound inducing several toxicity endpoints of interest. Using its ADMET evaluation module, compounds can be identified through their SMILES code, and information on the probability of toxicity in the endpoints selected can be processed by the server using deep learning models. For the present work, toxicity probabilities for the following endpoints were retrieved:

– FDA medium daily dose (FDAMDD), which provides an estimate of the toxic dose threshold of chemicals in humans;
– hERG blockers, related to the voltage-gated potassium channel encoded by hERG whose blockade may cause illness or even death;
– Hematotoxicity, which refers to the adverse effects of chemicals on blood-forming organs;
– Carcinogenicity, related to the ability to damage the genome or disrupt cellular metabolic processes;
– RPMI-8226 immunotoxicity, which helps determine the toxicity of compounds to a type of multiple myeloma cell line;
– Genotoxicity, which refers to the ability of harmful substances to damage genetic information in cells;
– Respiratory toxicity, which has become the main cause of drug withdrawal;
– A549 cytotoxicity, which helps determine the toxicity of compounds to a human non-small cell lung cancer cell line;
– Human hepatotoxicity, for compounds demonstrating adverse liver reactions;
– Drug-induced liver injury (DILI), for compounds that have well-known associations with liver injury and have a significant number (>10) of independent clinical reports of hepatotoxicity;
– Drug-induced nephrotoxicity, which refers to the harmful effects that occur in the kidneys;
– Hek293 cytotoxicity, which helps determine the toxicity of compounds to human embryonic kidney cells;
– AMES toxicity, which determines a mutagenic effect that has a close relationship with carcinogenicity;
– Skin sensitization, for potential adverse effects of dermally applied products;
– Eye irritation, which assesses the potential to affect cornea and conjunctiva tissues;
– Drug-induced neurotoxicity, for compounds that can harm both the central nervous system and the peripheral nervous system; and
– Ototoxicity, for compounds that have the potential to harm the inner ear by either damaging the ear’s structures directly or the nervous system.

### 4.8. Data Treatment and Statistical Analysis

Live and dead PWN counts were used to determine mortality percentages according to Formula (1):

(1) $$Mortality\;\left(\%\right)=\frac{dead\;PWNs}{total\;number\;of\;PWNs}\times 100$$

For the EO and compounds, mortality percentages were corrected to exclude control mortality through Formula (2):

(2) $$Corrected\;mortality\;\left(\%\right)=\frac{mortality\;in\;treatment\;\left(\%\right)-mortality\;in\;control\;\left(\%\right)}{100-mortality\;\left(\%\right)\;in\;control}\times 100$$

The toxicological strength of an EO or compound was characterized as complete when mortality was 100%, strong when above 80%, moderate when between 80 and 61%, weak when between 60 and 40%, and low or inactive when below 40% [31].

To determine half maximal effective concentration (EC_50_) values, the Origin Graphing and Analysis software, version 2019b, (OriginLab, Northampton, MA, USA) was used. A nonlinear regression analysis was performed by plotting corrected mortality values along EOs, fractions, or compound concentration values and fitting a dose–response log-logistic equation:

(3) $$y=A1+\frac{A2-A1}{1+10^{\left(EC50-x\right)p}}$$

where A1 and A2 are the lower and upper limits of the sigmoidal dose–response curve, respectively; *p* is the slope; and EC_50_ is the EO concentration that induces a response halfway between the lower and upper limits. The upper (A1) and lower (A2) limits were set to 0 and 100%, respectively. Determination of the lowest maximal effective concentration (EC_100_) was performed by solving the curve equation to the first y value of 100% mortality.

## 5. Conclusions

This study provides evidence for the use of *C. citriodora* EOs and their volatiles in sustainable PWN management. The principal EO constituents, citronellal and citronellol, demonstrated potent nematicidal activity while exhibiting a lower propensity for environmental and human health impacts compared to conventional pesticides. However, challenges remain in terms of the scalability and stability of EO-based formulations. With recent advancements in streamlining in vitro culture techniques, future research should prioritize optimizing the productivity of in vitro production systems, investigating synergistic interactions between EO components, and enhancing the stability and bioavailability of the bioactive compounds. Incorporating these biopesticides into integrated pest management strategies offers the potential to reduce the environmental footprint of plant protection products while ensuring the effective control of phytoparasitic nematodes.

## Figures and Tables

**Table 1:** Main constitutive volatile composition of *Corymbia citriodora* shoots (≥0.1%) from a 10-year-old mature tree (in vivo) and from in vitro shoot culture. Volatile profiles were obtained through a gas chromatography coupled with mass spectrometry (GC-MS) analysis of the essential oils.

Compounds	RT ^1^	RI ^1^	Relative Amount (%)	
			In Vivo	In Vitro ^2^
α-Pinene	7.46	930	1.3	0.5
Sabinene	8.75	958	t	0.2
β-Pinene	8.87	963	1.7	3.0
β-Myrcene	9.53	975	t	0.2
1,8-Cineole	10.87	1005	12.1	0.3
Limonene	10.98	1009	0.2	0.2
γ-Terpinene	12.22	1035	0.2	t
Linalool	13.88	1074	0.2	t
Isopentyl isovalerate	14.01	1084	0.1	
*trans*-Pinocarveol	14.29	1106	1.0	
Isopulegol	15.73	1116	14.2	1.5
Citronellal	16.16	1121	38.0	63.6
*neo*-Isopulegol	16.30	1121	1.0	0.7
*neoiso*-Isopulegol	16.76	1148	1.1	0.1
Terpinen-4-ol	17.13	1148	0.1	t
α-Terpineol	17.73	1159	0.4	t
Citronellol	19.81	1207	13.2	9.6
Geraniol	20.88	1236	t	0.1
Citronellyl acetate	25.53	1343	3.0	7.3
*trans*-β-Caryophyllene	28.65	1414	6.5	9.3
Aromadendrene	29.28	1428	0.3	
α-Humulene	30.02	1447	0.4	0.5
*allo*-Aromadendrene	30.21	1456	0.1	
Spathulenol	34.55	1551	0.2	
β-Caryophyllene oxide	34.87	1561	0.4	0.1
Globulol	34.98	1566	0.5	
Palmitic acid	46.80	1908		0.3
Phytol acetate	48.60	2101		1.0
Oleic acid	48.66	2119		0.1
Percentage of EO identification (%)			96.1	98.6
**Grouped components**				
Monoterpene hydrocarbons			3.5	4.1
Oxygen-containing monoterpenes			84.2	83.2
Sesquiterpene hydrocarbons			7.3	9.8
Oxygen-containing sesquiterpenes			1.2	0.1
Oxygen-containing diterpenes				1.0
Fatty acids				0.4

^1^ retention time (RT) and retention index (RI), relative to C_9_–C_22_ *n*-alkanes, on the DB-1 column, ^2^ volatile profile of *C. citriodora* shoots grown in vitro in Murashige and Skoog (MS) medium supplemented with 8 g/L of agar-agar, 30 g/L of sucrose, 0.5 mg/L of 6-benzylaminopurine (BAP), and 0.1 mg/L indole 3-butyric acid (IBA), t = trace.

**Table 2:** Corrected mortality percentages for the PWN exposed to 2 mg/mL of *Corymbia citriodora* essential oils or its main compounds, citronellal, citronellol, 1,8-cineole, isopulegol, and *trans*-β-caryophyllene, in comparison to the conventional nematicide emamectin benzoate. Mortality is presented as average ± standard error (%).

Compound	Chemical Class	Functional Group	Corrected Mortality (%)
*Corymbia citriodora* EO			98.1 ± 0.2
In vitro *C. citriodora* EO			100.0 ± 0.0
Citronellal	Oxygen-containing monoterpene	Aldehyde	100.0 ± 0.0
Citronellol	Oxygen-containing monoterpene	Alcohol	100.0 ± 0.0
1,8-Cineole	Oxygen-containing monoterpene	Ether	8.5 ± 0.2
Isopulegol	Oxygen-containing monoterpene	Alcohol	22.9 ± 0.9
*trans*-β-Caryophyllene	Sesquiterpene hydrocarbon	^1^	17.9 ± 0.6
Emamectin benzoate	Macrocyclic lactone	^2^	100.0 ± 0.0

^1^ hydrocarbon molecule, no functional group; ^2^ macrocyclic lactone with several functional groups.

**Table 3:** Nematicidal activity of *Corymbia citriodora* and in vitro *C. citriodora* shoot essential oils or their main compounds, the alcohol citronellol and its aldehyde citronellal, in comparison to emamectin benzoate, a conventional pesticide. The toxicological parameters presented are the concentrations needed to immobilize 20% (EC_20_), 50% (EC_50_), 80% (EC_80_) and 100% (EC_100_) of the sample population, based on dose–response curve fitting. The values for slope (*p*) and adjusted R^2^ are given for comparison purposes.

Parameter	*C. citriodora* EO	In Vitro *C. citriodora* EO	Citronellal	Citronellol	Emamectin Benzoate
EC_20_ (mg/mL) ^1^	0.677 ± 0.003	0.174 ± 0.009	0.186 ± 0.004	0.209 ± 0.002	0.170 ± 0.006
EC_50_ (mg/mL) ^1^	0.962 ± 0.005	0.239 ± 0.004	0.441 ± 0.008	0.307 ± 0.004	0.364 ± 0.009
EC_80_ (mg/mL) ^1^	1.247 ± 0.006	0.304 ± 0.011	0.696 ± 0.020	0.406 ± 0.007	0.557 ± 0.017
EC_100_ (mg/mL) ^2^	1.8 (1.6–1.9)	0.9 (0.4–1.0)	1.7 (1.6–1.8)	1.3 (1.0–1.3)	2.0 (1.7–2.0)
*p* ^1^	2.1 ± 0.2	9.3 ± 2.7	2.4 ± 0.1	6.1 ± 0.3	3.1 ± 0.1
Adj. R squared	0.985	0.993	0.978	0.988	0.971

^1^ the values are presented as average ± standard error; ^2^ the values are provided along with the upper and lower 95% confidence limits.

**Table 4:** Predicted environmental distribution (%) to the air, water, soil, and sediment environmental compartments using the Mackay fugacity model [17,18], predicted volatilization from water (using river and lake models) [18], estimated bioconcentration factor (BCF) and bioaccumulation factor (BAF) in aquatic environments [19], and projected removal through wastewater treatment of citronellal, citronellol, and the pesticide emamectin benzoate, determined through the EPI Suite™-Estimation Program Interface software [18] and based on the experimental chemical properties reported on online databases [20,21].

Compartment	Citronellal	Citronellol	Emamectin Benzoate
Air (%)	68.6	26.2	0.0
Water (%)	12.9	61.1	0.1
Soil (%)	18.1	12.4	97.6
Sediments (%)	0.4	0.3	2.2
Persistence (h)	263.0	392.0	10,400.0
Volatilization from water ^1^			
Model river (half-life in h)	4.0	14.2	1.1 × 10^6^
Model lake (half-life in h)	148.2	259.3	1.2 × 10^7^
Bioaccumulation estimates ^2^			
Bioconcentration factor (BCF) ^3^	82.5	67.7	5982.0
Bioaccumulation factor (BAF) ^3^	82.5	67.7	29,290.0
Biotransformation (half-life in days)	0.9	0.5	59.3
Removal in wastewater treatment ^4^			
Total removal (%)	15.9	8.4	93.9
Biodegradation (%)	0.1	0.1	0.8
Sludge adsorption (%)	5.2	5.5	93
Release to the air (%)	10.5	2.8	0.0

^1^ estimated through the WVOLWIN™ module for the rate of volatilization of a chemical from rivers and lakes [18]. ^2^ obtained through the BCFBAF™ module that provides estimates of the bioaccumulation factor (BAF) for generic fish species in the upper trophic level of aquatic food webs and the bioconcentration factor (BCF) for water exposure based on the Arnot–Gobas method [18,19]. ^3^ the values are obtained in L/kg FW (fresh weight). ^4^ estimated using the STPWIN™ module that predicts the percentage removal of a chemical in a typical activated sludge-based sewage treatment plant [18].

**Table 5:** The acute ecotoxicological thresholds (median lethal/effective concentration, LC_50_/EC_50_, mg/L) reported for citronellal, citronellol, or the pesticide emamectin benzoate against non-target aquatic model organisms (fish, algae, and invertebrates), retrieved from the ECHA [22], the PPDB (the Pesticide Properties Database) [21], and PubChem [20] online databases.

Compounds	Fish	Algae	Invertebrates ^5^
	LC_50_ (mg/L)	EC_50_ (mg/L)	EC_50_ (mg/L)
Citronellal	22.0 ^1^	13.3 ^3^	8.7
Citronellol	14.7 ^1^	2.4 ^3^	17.5
Emamectin benzoate	0.2 ^2^	0.001 ^4^	0.007

The values presented were reported for the fish species ^1^ *Leuciscus idus* or ^2^ *Oncorhynchus mykiss*, the algae ^3^ *Scenedesmus subspicatus* or ^4^ *Pseudokirchneriella subcapitata*, and the invertebrate ^5^ *Daphnia magna*.

**Table 6:** Experimental acute oral and dermal toxicity thresholds (mg/kg) retrieved from the PubChem [20], the PPDB (the Pesticide Properties Database) [21], and the ECHA [22] free online databases, and probabilities of acute toxicity for endpoints of human health predicted through the toxicity section of ADMETlab 3.0 online software tool [23] for citronellal, citronellol, and the conventional pesticide emamectin benzoate.

Experimental Thresholds	Citronellal	Citronellol	Emamectin Benzoate
Acute Dermal Toxicity (mg/kg)	2500 ^2^	2650 ^2^	439 ^1^
Acute Oral Toxicity (mg/kg)	2420 ^1^	3450 ^1^	82 ^1^
**Predicted Probability of Toxicity ^3^**			
A549 Cytotoxicity	0.057	0.078	0.086
AMES Mutagenicity	0.277	0.169	0.489
Carcinogenicity	0.301	0.449	0.076
Drug-Induced Liver Injury (DILI)	0.226	0.091	0.993
Drug-induced Nephrotoxicity	0.354	0.244	0.394
Drug-induced Neurotoxicity	0.524	0.354	0.855
Eye Irritation	0.996	0.998	0.000
FDA medium daily dose (FDAMDD)	0.079	0.049	0.744
Genotoxicity	0.012	0.001	0.933
Hek293 Cytotoxicity	0.033	0.038	0.512
Hematotoxicity	0.329	0.229	0.059
hERG Blockers	0.114	0.091	0.111
Human Hepatotoxicity	0.515	0.574	0.608
Ototoxicity	0.260	0.317	0.993
Respiratory Toxicity	0.647	0.612	0.111
RPMI-8226 Immunotoxicity	0.035	0.041	0.209
Skin Sensitization	0.964	0.963	0.326

The values presented were reported for ^1^ rat or ^2^ rabbit model organisms; ^3^ the values represent the probability of toxicity and vary between 0 and 1, with compounds displaying values lower than 0.3 being very unlikely toxic, those between 0.3 and 0.7 showing an intermediate probability of being toxic, and those higher than 0.7 are very likely to exhibit toxicity at each endpoint (underlined values). Access the online ADMETlab3.0 explanation page for a detailed explanation of each endpoint and specific toxicity [24].

**Table 7:** The physicochemical properties and partition coefficients of *Corymbia citriodora* essential oil main compounds citronellal and citronellol, and the pesticide emamectin benzoate compiled from the freely available online databases European Chemicals Agency (ECHA) [22], the PPDB (the Pesticide Properties Database) [21], and PubChem [20].

	Citronellal	Citronellol	Emamectin Benzoate
CAS number	106-23-0	106-22-9	155569-91-8
Molecular mass (g/mol)	154.3	156.3	1008.2
Melting point (°C)	−16.0	−20	146.0
Vapor pressure (Pa)	37.3	2.7	5.1 × 10^−6^
Solubility in water (mg/L)	88.0	307.0	24.0
Henry’s law constant (Pa.m^3^/mol)	26.4	2.1	1.7 × 10^−4^
logK_OW_ (Unitless)	3.5	3.9	5.0
K_OC_ (Unitless)	650.0	94.0	377,500

## Data Availability

The raw data is available from the corresponding author (Jorge M. S. Faria) upon reasonable request.

## Supplementary Materials

The following supporting information can be downloaded at: https://www.mdpi.com/article/10.3390/plants14131892/s1, Table S1: Nematicidal activity of *Corymbia citriodora* and in vitro *C. citriodora* shoots essential oils or their main compounds, the alcohol citronellol and its aldehyde citronellal, in comparison to emamectin benzoate, a conventional pesticide. The toxicological parameters presented are the concentrations needed to immobilize 50% (EC_50_) of the sample population, based on dose–response curve fitting.
