# Supplementary material for: In Vivo and In Vitro Grown Lemon-Scented Gum as a Source of Nematicidal Essential Oil Compounds
Source: Plants (Basel). 2025 Jun 20;14(13):1892. doi: 10.3390/plants14131892 (PMC12251783; doi:10.3390/plants14131892)
Supplement: Supplementary file 1 [file plants-14-01892-s001.zip › plants-3677158-supplementary.pdf]

plants

MDPI

Supplementary

In Vivo and In Vitro Grown Lemon-Scented Gum as a Source of Nematicidal Essential Oil Compounds

Jorge M. S. Faria [1,2,*], Gonçalo Pereira [1], Ana Cristina Figueiredo [3] and Pedro Barbosa [3,4]

[1] INIAV, I.P., National Institute for Agrarian and Veterinary Research, Quinta do Marquês, 2780-159 Oeiras, Portugal; goncalo.pereira@iniav.pt
[2] GREEN-IT Bioresources for Sustainability, Instituto de Tecnologia Química e Biológica, Universidade Nova de Lisboa (ITQB NOVA), Av. da República, 2780-157 Oeiras, Portugal
[3] CE3C, Centre for Ecology, Evolution and Environmental Changes & CHANGE—Global Change and Sustainability Institute, Faculdade de Ciências da Universidade de Lisboa, DBV, C2, Campo Grande, 1749-016 Lisboa, Portugal; acsf@fc.ul.pt (A.C.F.); pbarbosa@uevora.pt (P.B.)
[4] MED, Mediterranean Institute for Agriculture, Environment and Development & CHANGE—Global Change and Sustainability Institute, Institute for Advanced Studies and Research, Évora University, Pólo da Mitra, Ap. 94, 7006-554 Évora, Portugal
* Correspondence: fariajms@gmail.com

Table S1. Nematicidal activity of *Corymbia citriodora* and *in vitro C. citriodora* shoots essential oils or their main compounds, the alcohol citronellol and its aldehyde citronellal, in comparison to emamectin benzoate, a conventional pesticide. The toxicological parameters presented are the concentrations needed to immobilize 50 % (EC_{50}) of the sample population, based on dose–response curve fitting.

Parameter	*C. citriodora* EO	*In vitro C. citriodora* EO	Citronellal	Citronellol	Emamectin benzoate
EC_{50} (mg/mL)[1]	0.962 (0.953–0.969)	0.239 (0.098–0.248)	0.441 (0.426–0.456)	0.307 (0.300–0.314)	0.364 (0.358–0.368)

[1] – the values are provided along with the upper and lower 95% confidence limits.
